# Supplementary material for: Genomic hotspots in the DENV-2 serotype (E, NS4B, and NS5 genes) are associated with dengue disease severity in the endemic region of India
Source: PLoS Negl Trop Dis. 2025 Apr 29;19(4):e0013034. doi: 10.1371/journal.pntd.0013034 (PMC12040166; doi:10.1371/journal.pntd.0013034)
Supplement: S1 Fig — (PDF) [file pntd.0013034.s011.pdf]

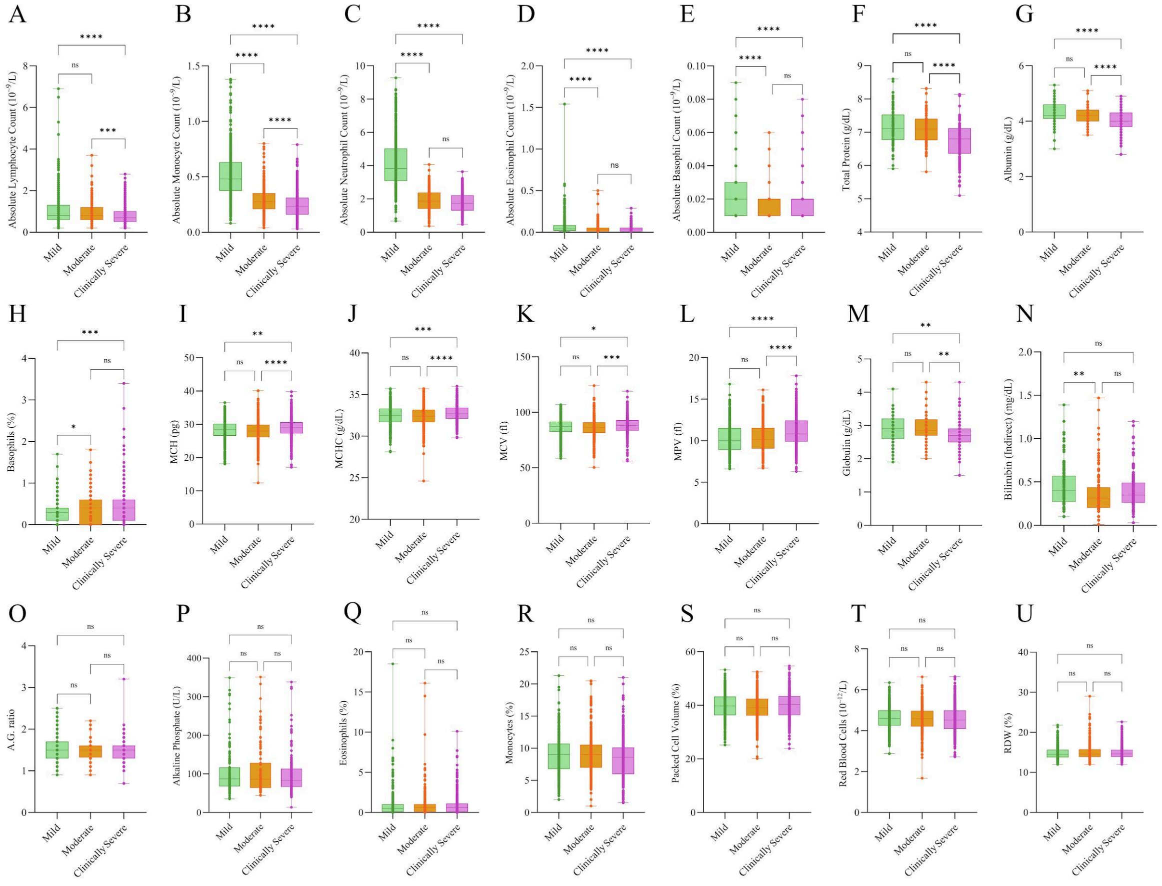

S1 Fig. Statistical analysis of CBC and LFT parameters across mild, moderate, and clinically severe patients. Statistical comparison across mild, moderate, and clinically severe dengue patients for the (A) Absolute Lymphocyte Count (10^9/L); (B) Absolute Monocyte Count (10^9/L); (C) Absolute Neutrophil Count (10^9/L); (D) Absolute Eosinophil Count (10^9/L); (E) Absolute Basophil Count (10^9/L); (F) Total Protein (g/dL) (G) Albumin (g/dL); (H) Basophils (%); (I) MCH (pg); (J) MCHC (pg); (K) MCV (fl); (L) MPV (fl); (M) Globulin (g/dL); (N) Bilirubin (Indirect) (mg/dL); (O) A.G. ratio; (P) Alkaline Phosphatase; (Q) Eosinophils (%) (R) Monocytes (%); (S) Packed Cell Volume (PCV); (T) Red Blood Cells (10^{12}/L) and (U) RDW (%). Kruskal-Wallis with Dunn's comparison was performed for comparison of each clinical parameter across mild, moderate, and clinically severe. The significance value is denoted as *, where * indicates $p \leq 0.05$, ** indicates $p \leq 0.01$, *** indicates $p \leq 0.001$, and **** indicates $p \leq 0.0001$.
