# Supplementary material for: Genomic hotspots in the DENV-2 serotype (E, NS4B, and NS5 genes) are associated with dengue disease severity in the endemic region of India
Source: PLoS Negl Trop Dis. 2025 Apr 29;19(4):e0013034. doi: 10.1371/journal.pntd.0013034 (PMC12040166; doi:10.1371/journal.pntd.0013034)
Supplement: Attachment — Submitted filename: Response to Reviewers.docx [file pntd.0013034.s013.docx]

**Reviewers' Comments:**

**Reviewer #1:**

The article by Ravi et al. adressess an important research question, as to whether genetic variation in the DENV-2 genome is associated with an increased risk of developing severe complications during an infection with the virus. The sample size is large and the clinical characterization of the patient goups should in theory be sufficient to detect genetic variation that may influenze disease severity. However, there are a few issues and limitations that may be adressed in order to better support the findings and highlight the strengths of the study:

We thank the Reviewer for dedicating the time to review our manuscript thoroughly. We truly appreciate your positive feedback on the research concept and the research question. Your insights are invaluable to us. Our point-by-point responses to your comments are as follows:

1. It needs to be made clear that there was not a single severe dengue case in this study, and the use of the term severe dengue should be clarified, that the study uses this as a self-defined category and refers to pacients with both, thrombocytopenia+leukopenia

We appreciate the reviewer’s observation. Although we have described the severity classification in the methodology of the original submission under the section “study design and sample processing”, in the revised submission we have replaced the term “severe” with “clinically severe” dengue subgroup (Thrombocytopenia+leukopenia) throughout the manuscript. Additionally, a table outlining the subgroup definition is included in the revised submission.

2. Patient sample sizes should be mentioned in the results and abstract

We acknowledge the reviewer for the suggestion. In response to the reviewer suggestion, we have now mentioned the sample number in the revised abstract. The revised abstract has been appended here for the easy perusal of the reviewer. All numbers about the sample size, such as total serum samples (4045), number of RT-PCR conducted (4045), number of each serotype of DENV identified (DENV1=28, DENV2=3702, DENV3=45, DENV4=05, DENV negative=265), number of DENV-2 genome sequenced (3702) etc. are mentioned in the result section.

ABSTRACT:

*“Dengue virus (DENV) infection manifests a wide spectrum of clinical outcomes, ranging from mild fever to severe and potentially fatal disease, yet the factors driving this variability remain poorly understood. This study aims to unravel the relationship between clinical manifestations of dengue and the genetic diversity of the virus, providing insights into the genomic variability driving disease severity. To achieve this, serum samples were collected during a dengue outbreak in National Capital Region-Delhi, India, from June to November 2023. Serotyping of RNA isolated from 4,045 clinical serum samples revealed DENV-2 as the predominant serotype in circulation (n=3702). Whole-genome sequencing for 3702 clinical samples was performed using Oxford Nanopore Technology (ONT) further yielding 3254 DENV-2 genomes with >50% coverage. However, all of them identified the cosmopolitan genotype of DENV-2, forming a distinct monophyletic cluster in the global phylogenetic tree. Comprehensive variant analysis uncovered 1,618,158 single nucleotide variations (SNVs) across the sequenced DENV-2 population. The clinico-genomic approach carried out in 1294 samples, mild (n=473), moderate (n=405) and clinically severe (416), reveals a significant burden of SNVs in various genomic regions linked to differential disease outcomes. Statistical analyses, including Fisher’s exact test and phi-correlation, identified hotspot regions in the Envelope (E), NS4B, and NS5 genes, where SNVs were strongly associated with mild and clinically severe phenotypes, providing insights into the genomic determinants of disease severity. Interestingly, the clustering of severity-associated SNVs in these genomic hotspot regions highlights their potential as therapeutic targets within the DENV genome. These findings offer a promising direction for developing early mitigation strategies and targeted interventions to manage the progression of severe DENV infections.”*

3. Table 1: Please provide sample size of patients in table.

We thank the reviewer for the suggestion. We have now included the sample size for each parameter in **Table 1.** With additional information and clarity, we have replaced **Table 1** with **Supplementary Table 9** in the revised manuscript.

4. It is known that mutations in the 3'UTR of the viral genome, a region that codes for sfRNAs is relevant for viral pathogenesis and immune evasion.The following two publications provide the evidence for that and should be included in the discussion section of the article:
   <https://www.ncbi.nlm.nih.gov/pmc/articles/PMC6545344/>
   <https://pubmed.ncbi.nlm.nih.gov/26138103/>
   I would suggest that the authors specifically looked in more detail for mutations in the relevant regions in the 3'UTR, as they found a significant difference in frequencies of SNVs in the 3'UTR when comparing mild and severe groups (See results section: lines 346 and 351).

We thank the reviewer for the suggestion. In our study, we got two significant mutations in the 3'UTR, one associated with mild (A10424G) and one with clinically severe (G10296A) sub-phenotype. We have now mentioned their potential functional relevance in the discussion section of the revised manuscript, mentioned below for the easy perusal of the reviewer.

*“While SNVs in the 3′UTR and their epidemiological roles are well studied, our findings highlight distinct evolutionary pressures in dengue virus adaptation. The mild fNR2 (flaviviral nuclease-resistant RNA) mutation (A10424G) helps maintain sfRNA (subgenomic flavivirus RNA) stability for efficient transmission (*[*https://doi.org/10.1016/j.isci.2019.05.019*](https://doi.org/10.1016/j.isci.2019.05.019)*), while the clinically severe sfRNA mutation (G10296A) may enhance immune evasion by altering interactions with the host factors like TRIM25 (*[*https://doi.org/10.1126/science.aab3369*](https://doi.org/10.1126/science.aab3369)*). This suggests a potential balance between viral fitness and immune adaptation, influencing disease severity.”*

5. It is not clear how the authors categorized SNVs in the untranslated regions of the Dengue genome into synonymous and non-synonymous SNVs (mainly Supplementary Table 6). Please clarify.

We thank the reviewer for the query. In the untranslated regions (UTRs) of the Dengue virus genome, all mutations are inherently synonymous as they do not undergo translation. However, for our analysis and futuristic implications (yet to be understood), we evaluated whether the change in the DNA base could have theoretical potential to alter the amino acid sequence? And thus, we categorized SNVs in the 3'UTR as synonymous or non-synonymous based on the codon from which the mutation occurred. This is because UTR mutations can influence overlapping open reading frames (ORFs), which play a crucial role in translation.

6. The definitions for mild, severe and moderate Dengue are critical for the entire analysis in this study. This is only mentioned in the methods section. There is a high risk of confusing this with the clinical term of "severe dengue" as based on clincial severe symptoms (see <https://www.who.int/emergencies/outbreak-toolkit/disease-outbreak-toolboxes/dengue-outbreak-toolbox>). I suggest to use the definition as explained in lines 545ff throughout the manuscript in order to make clear which groups were analyzed here. Please include sample sizes as well.

We appreciate the reviewers’ suggestion. To avoid any potential confusion from the reader's perspective, we have explicitly mentioned about the clinically severe phenotype throughout the manuscript. Additionally, in response to your suggestion and that of another reviewer, we have included a brief table outlining the definitions of the mild, moderate, and clinically severe subgroups, along with the sample size for each subgroup in the revised submission.

Still to make it even more explicit, we have now used “clinically severe dengue” in the revised manuscript instead of “severe dengue” to make distinction from the WHO one.

7. In terms of disease progression it is important to know at which time point after onset of disease the blood parameter were assessed. As it may be possible that a patient at time of enrolement was categorized as mild or moderate, may later develop thrombocytopenia and leukopenia and fall the "severe group". How can be made sure that this is not the case? Do the authors have any additional information on the outcome of the infection in the enrolled patients?

We thank the reviewer’s inquiry and take this opportunity to clarify an important perspective. Samples were collected from all the dengue infected patients at the time of admission to the hospital and based on their clinical parameters during enrolment, the patients were further categorized into mild, moderate, and clinically severe subgroups. As mentioned in the manuscript, one of the premises of this work is to see whether when dengue patients report to a hospital, does dengue virus genomes (vis-à-vis genomic mutations) hold clues towards their role in the potential disease severity sub-phenotypes. Incidentally, we did not have any information on the outcome of the infection in the dengue patients. But yes, future longitudinal studies may help us in better understanding of the dengue pathogenesis with known clinical outcome.

8. Lines 462ff: The authors state "Critically, the significant alterations observed in hematological and hepatic parameters across the dengue patients highlight their potential as diagnostic and prognostic biomarkers for severe dengue". The study itself has the potential to do so, and a result may be a list of these biomarkers. I recommend listing them, however, not using the term "severe dengue" as it might be missleading, but instead try to provide biomarkers for thrombocytopenia and leukopenia. Ideally create new subgroups and compare level of hematological and hepatic parameters between the following groups of patients: none (Reference group), thrombocytopenia only, leukopenia only, thrombocytopenia + leukopenia.

We thank the reviewer for the valuable suggestions. As suggested by you, we have compared the hematological and hepatic parameters across the patients subgroup namely: Reference (no leukopenia, no thrombocytopenia); only leukopenia; only thrombocytopenia; and both leukopenia and thrombocytopenia. We also performed Kruskal Wallis test with Dunn’s comparison for multiple pairwise comparison across each hematological and hepatic parameters. The median and significance from the comparative analysis is mentioned in the **table**. Hematological parameters across all groups remain within the reference range, implying that while alterations exist, they may not serve as definitive severity indicators. The liver enzyme, AST level observed significantly high in the dengue patients group having thrombocytopenia and both thrombocytopenia and leukopenia, suggesting AST level in dengue patient could potentially be used as the prognostic marker for both thrombocytopenia and leukopenia.

Table: Comparison of clinical parameters between the Reference group, Thrombocytopenia, Leukopenia and Combined Thrombocytopenia-Leukopenia

| Group | Reference vs. Leukopenia | | | Reference vs. Thrombocytopenia | | Reference vs. Leukopenia + Thrombocytopenia | |
| --- | --- | --- | --- | --- | --- | --- | --- |
|  | Reference | Leukopenia | Sig | Thrombocytopenia | Sig | Leukopenia + Thrombocytopenia | Sig |
| TLC (10^9^/L) | 5.4 | 3.2 | **** | 5 | ns | 2.9 | **** |
| Platelet (10^9^/L) | 180 | 165 | *** | 105 | **** | 103 | **** |
| NS1 | 4.5 | 4.14 | ns | 3.8 | ns | 5.165 | ns |
| Haemoglobin (g/dL) | 13 | 12.7 | ns | 13.6 | *** | 13.2 | ns |
| Packed Cell Volume (%) | 39.75 | 39.1 | ns | 41.25 | ** | 40.3 | ns |
| RBC (10^12^/L) | 4.625 | 4.595 | ns | 4.68 | ns | 4.535 | ns |
| MCV (fL) | 87.35 | 86.3 | ns | 89.15 | ** | 88.3 | ns |
| MCH (pg) | 28.5 | 27.95 | ns | 29.2 | *** | 28.9 | ** |
| MCHC (g/dL) | 32.5 | 32.4 | ns | 32.7 | ** | 32.7 | ** |
| MPV (fL) | 10 | 10.1 | ns | 11.1 | **** | 10.92 | **** |
| RDW (%) | 14.5 | 14.6 | ns | 14.8 | * | 14.6 | ns |
| Neutrophils (%) | 73.9 | 60 | **** | 66.65 | **** | 61.65 | **** |
| Lymphocytes (%) | 15.05 | 28.9 | **** | 22 | **** | 28.325 | **** |
| Monocytes (%) | 9 | 9.1 | ns | 9.6 | ns | 8.6 | ns |
| Eosinophils (%) | 0.5 | 0.5 | ns | 1 | ns | 0.6 | ns |
| Basophils (%) | 0.3 | 0.4 | * | 0.3 | ns | 0.4 | *** |
| Absolute Neutrophil Count (10^9^/L) | 3.83 | 1.87 | **** | 3.255 | *** | 1.725 | **** |
| Absolute Lymphocyte Count (10^9^/L) | 0.8 | 0.9 | ns | 1.2 | **** | 0.7 | **** |
| Absolute Monocyte Count (10^9^/L) | 0.48 | 0.28 | **** | 0.48 | ns | 0.23 | **** |
| Absolute Eosinophil Count (10^9^/L) | 0.04 | 0.03 | **** | 0.05 | ns | 0.03 | **** |
| Absolute Basophil Count (10^9^/L) | 0.02 | 0.01 | **** | 0.02 | ns | 0.01 | **** |
| Total Protein (g/dL) | 7.1 | 7.075 | ns | 6.67 | **** | 6.8 | **** |
| Albumin (g/dL) | 4.2 | 4.2 | ns | 3.9 | **** | 4 | **** |
| Globulin (g/dL) | 2.9 | 2.8 | ns | 2.8 | ns | 2.7 | * |
| A.G. | 1.5 | 1.5 | ns | 1.4 | * | 1.5 | ns |
| Bilirubin (Total) (mg/dL) | 0.5 | 0.4 | *** | 0.7 | *** | 0.55 | ns |
| Bilirubin (Direct) (mg/dL) | 0.15 | 0.12 | ns | 0.2 | *** | 0.18 | ns |
| Bilirubin (Indirect) (mg/dL) | 0.39 | 0.3 | ** | 0.49 | * | 0.36 | ns |
| Transaminase (AST) (U/L) | 41 | 52.8 | ** | 110.6 | **** | 90.3 | **** |
| Transaminase (ALT) (U/L) | 35 | 37 | ns | 72 | **** | 56.1 | **** |
| Alkaline Phosphatase (U/L) | 87 | 85 | ns | 91 | ns | 84 | ns |
| GGTP (Gamma GT), Serum (U/L) | 27 | 22.95 | ns | 54.4 | **** | 35 | ns |

**Reviewer #2:**

Ravi et al. collected 4045 serum samples of DENV-NS1-antigen positive and found 3702 DENV-2. Based on 3254 DENV-2 E region sequences and some of full -length sequences, the authors detected nucleotide polymorphisms.The main finding should be that the sequence variation which is related to disease severity from the title of this manuscript, but it is not clear due to the lack of functional analysis of mutated virus.

1. The authors display the number of SNV, but the specific position or amino acid variation is more important. In this manuscript, the data presentation should be more focused on the chapter “Distinct Genomic Hotspots in E, NS4B and NS5 Regions Underpin Dengue Disease Severity”. In this version, it is not easy to specify the position which is related to disease severity.

We acknowledge the reviewer's query. We have now included the following section in the result section to have more position specific or amino acid variation in the revised manuscript,

*“Our domain-wise analysis of SNVs in the E-gene reveals a clear demarcation in the distribution of clinically severe and mild mutations, highlighting their potential functional significance in disease severity. Clinically severe mutations predominantly occur in the DI-DIII region [A1335G, G1374A, G1503A, C1713T, A1830G, A2016G,* and *C2026T], which is critical for receptor binding, membrane fusion, and viral entry (*[*https://doi.org/10.1128/JVI.00224-12*](https://doi.org/10.1128/JVI.00224-12)*). In contrast, mutations associated with mild sub-phenotype are localized in the DIII and stem regions [C2073T, G2085A, A2105G, T2124C, G2157A,* and *G2160A], which are primarily involved in the virus assembly and host cell recognition (*[*https://doi.org/10.1128/jvi.02099-10*](https://doi.org/10.1128/jvi.02099-10)*). Additionally, in the NS4B and NS5 regions, we observe a mixture of SNV associations, suggesting diverse functional impacts on viral replication, immune modulation, and overall disease progression.”*

2. In line 318, the authors said that “The frequency of the SNVs across the mild, moderate, and severe is depicted in Figure 4D“. It should be shown in Table. The supplemental Tables should show the frequency but not number in three groups, mild, moderate and severe and ANOVA test are required. The escalation of the frequency from mild, moderate, to severe cases will be shown on the responsible SNVs.

We appreciate the reviewer’s valuable suggestion. As correctly pointed out, we have provided the mutation count distribution across the virus population for mild, moderate, and clinically severe cases in our **S4 Table**. We have updated the frequency of the mutation in the **S4 Table** of the revised manuscript. Additionally, in response to the reviewer’s suggestion we have done gene-wise ANOVA to see the frequency escalation from mild to clinically severe sub-phenotype (Table below), we have obtained the following results,

**Table:** Frequency escalation and ANOVA results (F-statistic & P-value) comparing the mean frequencies of mild, moderate and clinically severe groups

| **Gene** | **Mild Frequency (%)** | **Moderate**  **Frequency (%)** | **Clinically Severe**  **Frequency (%)** | **F-statistic** | **P-value** |
| --- | --- | --- | --- | --- | --- |
| 5’UTR | 11.9 | 11.3 | 10.8 | 0.004 | 0.9 |
| C | 18.9 | 18.1 | 17.6 | 0.02 | 0.9 |
| ancC | 24.4 | 24.5 | 24.7 | 0.0002 | 0.9 |
| M | 16 | 15.9 | 15.8 | 0.0007 | 0.9 |
| prM | 23.2 | 23.4 | 23.7 | 0.002 | 0.9 |
| E | 20.5 | 20.7 | 20.6 | 0.02 | 0.9 |
| NS1 | 20.2 | 20.4 | 20.3 | 0.001 | 0.9 |
| NS2A | 23.3 | 24 | 24 | 0.01 | 0.9 |
| NS2B | 23.3 | 24 | 24 | 0.03 | 0.9 |
| NS3 | 24.4 | 24.4 | 24 | 0.03 | 0.9 |
| NS4A | 17 | 15.9 | 15.5 | 0.06 | 0.9 |
| NS4B | 20.2 | 20 | 19.5 | 0.02 | 0.9 |
| NS5 | 19.3 | 19.4 | 19.2 | 0.004 | 0.9 |
| 3’UTR | 5.6 | 5.5 | 5.8 | 0.009 | 0.9 |

- Our P-value is insignificant and F-statistic for all the genes came very low indicating there is little variance between the severities within every gene.
- This indicates the frequency differences between mild, moderate, and clinically severe cases are minimal, making the mean mutation frequency potentially an unsuitable metric for capturing the differences between the severity subgroups. This is possibly due to the slow evolutionary rate of dengue viruses; the observed severity differences arise within the same infection period (June to November 2023). This reinforces the need for a categorical SNV distribution approach, which we have implemented in our further analysis to better capture the relationship between specific mutations and disease severity.

3. The Figure 5 must be more conclusive, which display only the position which is related to disease severity. The data presentation of Figure 5 should be re-considered to merge mild, and severe in one line to compare these groups.

We thank the reviewer for the suggestion. We have merged the mild and clinically severe in one line and updated a new figure in the revised manuscript.

**Fig 5. Distribution of significant SNVs across disease severity.** Lollipop plot depicting significant SNVs across mild and clinically severe. The SNV is represented with different colors, signifying individual groups. Significance was calculated using Fisher’s exact test (p<0.05). The directionality and strength of association of significant SNVs with respective groups were confirmed with the phi coefficient correlation test.

4. It is not easy to understand the definition of mild, moderate and severe. The table of criteria should be included. It should be noted that the disease severity of dengue cases are usually determined by clinical symptoms but not the laboratory data.

We are thankful to the reviewer’s suggestions. For an easy perusal, in the revised manuscript, we have included a **Table 1**, which tabulates the criteria used to define mild, moderate, and clinically severe dengue groupings.

**Table 1.** Criteria based on clinical parameters for defining mild, moderate, and clinically severe dengue patients

| **Group** | **Total leukocyte count** | **Platelet count** |
| --- | --- | --- |
| Mild (n=473) | Normal | Normal |
| Moderate (n=405) | Decreased (leukopenia) | Normal |
| Clinically Severe (n=416) | Decreased (leukopenia) | Decreased (thrombocytopenia) |

During dengue infection, clinical symptoms are considered to define the severity of infection. Fluctuations across certain clinical parameters including total leukocyte count (TLC) and platelet count are known to be associated with dengue patients and are considered as major predictive markers for dengue fever (DF) (<https://doi.org/10.7759/cureus.11594>). Prolonged leukopenia (reduced TLC) and thrombocytopenia (reduced platelet count) have been implicated in the transition from mild to severe dengue, likely due to their role in impairing the host's immune response and exacerbating vascular leakage, bleeding tendencies, and organ failure (<https://doi.org/10.1371/journal.pntd.0007837>, <https://doi.org/10.1371/journal.pntd.0009692>). This decrease in leukocytes and thrombocytes compromises the immune system's ability to combat secondary infections (<https://doi.org/10.1155/2015/313842>). Therefore, we selected these two parameters as primary criteria for categorizing dengue patients into mild, moderate, and clinically severe sub groups.

5. In Figure 2, the color of Mild (M), moderate (MD), Severe (SV) are not distinguishable. It should be modified. For example, decrease the number of reference sequence from the world and enlarge the part of in-house.

We thank the reviewer for the suggestion. We have rebuilt one more phylogeny by taking only mild (n=364), moderate (n=280) and clinically severe (n=285) to get visually clear clades of our disease sub-phenotypes, which is updated in the revised manuscript as **Fig 3B**.

**Fig 3B:** A magnified view of phylogenetic tree for 929 genomes comprising clinical sub-phenotypes mild (light green), moderate (orange), and clinically severe (magenta).

6. In line 558, how many sequences were successfully determined whole genome sequence?

Thank you for your query. Out of 3702 sequenced DENV-2 genomes, we achieved 95-98% genome coverage for 520 genomes, and 90-95% coverage for 1668 genomes. In total 2188 DENV-2 genomes exceeded 90% coverage, representing the substantial number of DENV-2 population under the study. Importantly, the genomes were sequenced directly from clinical serum samples which could result in differential genome coverage. The sequencing output is likely to be a reflection of RNA copies and its integrity into the clinical serum samples- as these two factors could influence the genome coverage of DENV-2 population sequenced using the RNA directly isolated from the clinical samples.

**Reviewer #3:**

The manuscript presents a robust clinico-genomic study on DENV-2 with an impressive sample size and advanced sequencing techniques. The methodology is well-structured, but some sections could benefit from additional details for reproducibility. Some sections, particularly Results and Conclusions, require tighter integration of findings with broader implications.

1. The ethics approval and consent process are mentioned (Page 10, Line 516–519). However, consider including specific details about patient inclusion/exclusion criteria.

Thank you Reviewer for the suggestion. Initially, we started with the patients tested positive for NS1-Ag in dengue. Subsequently, the NS1-Ag results were validated using serotype-specific RT-PCR. The RT-PCR findings indicated that the majority of patients were infected with DENV-2 (3702 out of 4045 patients). Thus, “combined positives from NS1 and RT-PCR have been used in the study”. This we have stated now in the revised manuscript in the section title “DENV-2: A predominant serotype in circulation”

2. On Page 11 (Lines 520–531), the description of sample storage is clear. Adding information about how serum integrity was verified post-transportation would improve this section.

Thank you for your valuable suggestion. To ensure the integrity of the serum samples, we have implemented several precautions, which we would like to highlight:

- The serum samples were collected daily from patients at the hospital and transported to our laboratory within the same city in cold storage, with transportation times ranging from 45 to 60 minutes only. Upon arrival, the samples were aseptically transferred into sterile 2 ml tubes, with the transfer process carried out on ice to preserve sample integrity.
- The serum samples were processed for RNA isolation on the same day for better possibility of good integrity RNA. Any remaining serum samples were stored at -80°C for long-term preservation (in case required). RNA integrity was verified through agarose gel electrophoresis, while RNA quantity and quality were further assessed using a Nanodrop spectrophotometer.

To enrich this information, we have edited the “study design and sample processing” section in the revised manuscript. Pasting the paragraph showing the edited text below for the perusal of reviewer

*“A total of 4045 NS1-Ag-positive serum samples were brought to our laboratory at CSIR-Institute of Genomic and Integrative Biology (CSIR-IGIB), New Delhi, India, in a cold storage box on a daily basis during the study period. The transportation time was ranging from 45 to 60 minutes. On the same day, the serum samples were aseptically transferred to the 2 ml tubes and processed for the RNA isolation the same day. Any remaining serum samples were stored at -80°C for long-term preservation (in case required)”*

3. On Page 14 (Lines 550–556), the statistical methods used are appropriate. However, clarify whether any corrections for multiple comparisons were applied during the analysis.

We acknowledge the reviewer’s query. Yes, we have performed multiple comparisons and applied appropriate corrections when analyzing clinical parameters in the results section, *"Significant Alterations in the TLC, Platelets, and Liver Enzymes across DENV-2 Infected-Patients".* Specifically, we used the Kruskal-Wallis test for multiple comparisons and applied Dunn’s correction to account for statistical significance. This we have stated in the same section and also in **Fig 3** of the revised manuscript.

4. For the whole-genome sequencing (Page 13, Lines 540–549), provide specifics about sequencing depth and coverage uniformity, as these are critical for assessing data quality.

We thank the reviewer for the suggestion. In alignment with the reviewer’s suggestion, we have provided a detailed summary of sequencing depth and genome coverage for each sample in the **S2 Table**. Furthermore, the SNV count and corresponding coverage details are tabulated in **S5 Table**. In addition to that, we have added the following paragraph in the revised manuscript,

*“We have generated a significantly high number of sequencing reads, achieving a highest read of 2,23,095, with average depth of 1081X and an average genome coverage of 87.5 per Dengue virus genome. The highest genome coverage obtained was 98.8, which is commendable for direct clinical sample sequencing at a large scale. For basecalling and barcode demultiplexing, we utilized the dorado(v7.2) with the "hac" (high-accuracy basecallingl) algorithm. This algorithm enhances sequencing accuracy by using a deep-learning model trained to minimize basecalling errors, ensuring high-fidelity read conversion from raw nanopore signals. Furthermore, Variant calling were carried out using Clair3, a deep-learning-based variant caller specifically designed for long-read sequencing. To ensure high-quality variant identification with uniform depth of coverage, we applied a stringent read-depth threshold, masking variants with depth below 20X.”*

5. While the manuscript mentions 4045 samples (Page 11, Line 526), justify the sample size for statistical robustness and clinical relevance.

We acknowledge the reviewer’s important query and clarify the same. Out of 4,045 samples, we had detailed clinical data for various parameters for a subset of this master sample set, which is 1294 samples. We conducted an integrative clinico-genomic analysis on these 1,294 samples, categorizing them into mild (n=473), moderate (n=405), and clinically severe (n=416) sub-groups. All our clinical and genomic analyses were performed based on comparisons within these subgroups. Given our matching sample size within each subgroup, statistical robustness has been maintained, ensuring that the findings are both clinically relevant and well integrated with future implications.

6. Expand on data normalization or preprocessing steps for variant analysis.

We thank the reviewer for the suggestion. To ensure high-quality variant analysis, we implemented rigorous data preprocessing and normalization steps. Initially, variant calling was performed using Clair3, a deep-learning-based variant caller optimized for long-read sequencing, which improves SNV and indel detection through a pileup and full-alignment approach. After variant calling, we conducted post-VCF analysis, applying a genotype quality filter (>3) and depth filter (>20) to retain reliable variants. We have mentioned the same in the methodology section of the manuscript. In response to the reviewer's comments, we have incorporated the additional variant preprocessing steps prior to annotation of the variants, into the revised manuscript. Providing them here for the reviewer’s easy reference.

*“After merging the multiple VCFs, the preprocessing steps involve* ***‘IndelGap’*** *correction and* ***‘multiallelic’*** *variant resolution using bcftools, allowing us to extract a single variant from a single position. To facilitate downstream analysis, we applied variant normalization using* ***‘norm’*** *and* ***‘sort’****, ensuring proper merging of mutations and maintaining a consistent positional order from start to end.”*

7. Figures and tables are detailed, but some captions lack clarity. For instance, Figure 1 (Page 17) needs an expanded description to guide interpretation.

We thank the reviewer for the suggestion, we have given more guidance for the Figure-1 caption in the revised manuscript, sharing it here for the easy perusal of the reviewer.

***“Figure 1. Experimental workflow and SNVs spectrum across DENV-2 genomes.*** ***(A)*** *Study design includes NS1-Ag-positive serum sample collection, RNA isolation, and serotype detection, followed by whole genome sequencing, variant calling, and downstream data interpretation. This figure was created using Biorender.com.* ***(B)*** *SNV spectrum with distribution of synonymous and non-synonymous SNVs across DENV-2 genomes whose coverage are >50% (n=3254).* ***(C-D)*** *Bar chart representing means of number of unique mutation divided by gene length, a normalized count in the y-axis, with bootstrap standard error estimation* ***(C)*** *synonymous, and* ***(D)*** *non-synonymous SNV counts (x1000) across the DENV-2 genomes.* ***(E)*** *Stacked bar chart depicts the percentage proportion of synonymous and various types of non-synonymous SNVs—including missense, frameshift, and stop-gained—across different genes of DENV-2 genomes.”*

We have also revisited the figure legends of other figures and made necessary updates, were required.

8. Results on Page 19 (Lines 600–610) describing SNV distribution could be better supported by including a visual density plot.

We acknowledge the reviewer's suggestion to enhance the SNV distribution results with a visual density plot. However, the context highlighted and referenced Page 19 (Lines 600–610) indicated by the reviewer does not match, apparently making it difficult for us to include the suggestion. Additionally, for SNV distribution, we have referenced Figure 1B, Figure 4D, and Figure 5 in the manuscript.

Hopefully, that accounts for the visual plot towards SNV.

9. The identification of hotspot regions in NS5 and E genes (Page 22, Line 645) is significant but requires contextualization. How do these compare with findings from other regions or similar studies?

Thank you for the suggestion. We have incorporated the following paragraph in the discussion section of the manuscript.

*“Prior to this study, few investigations have linked SNVs in the E and NS5 regions of DENV-2 to disease severity. For example, an isoleucine-to-valine substitution at position 322 in EDIII of the E gene has been associated with both dengue fever (DF) and dengue hemorrhagic fever (DHF) (*[*https://doi.org/10.1371/journal.pone.0121696*](https://doi.org/10.1371/journal.pone.0121696)*). Similarly, the Gly605Val mutation in NS5 has been linked to increased virulence and severe dengue outcomes (*[*https://doi.org/10.1371/journal.pone.0054220*](https://doi.org/10.1371/journal.pone.0054220)*), while T7812G (G81G) and C9420A (A617A) substitutions are thought to enhance viral replication (*[*https://doi.org/10.3389/fmicb.2022.894200*](https://doi.org/10.3389/fmicb.2022.894200)*). However, these studies have mostly focused on individual mutations and have not fully captured the genetic diversity across the DENV-2 populations.”*

This highlights the need for large-scale clinico-genomic studies in dengue endemic regions like India. Unlike prior work, we analyzed a diverse DENV-2 population and identified SNV hotspots in the E and NS5 regions significantly associated with the dengue severity, offering new insights into the virus-host dynamics and the molecular mechanisms underlying disease severity sub-phenotypes in the regions with significant dengue disease load.

10. Page 23 (Lines 670–675) mentions clustering of severe-associated SNVs in specific genomic regions. Highlight potential functional implications or experimental validations, if any.

We acknowledge the limitation of experimental validation for SNVs in DENV. However, we have compared SNV frequencies with data from other countries and provided these updates in **Figure 6** for broader context. Also, we have cited a few experimental studies in the discussion of the revised manuscript, attaching it here for the easy perusal of the reviewer.

*“The Gly605Val mutation in NS5 has been linked to increased virulence and severe dengue outcomes (*[*https://doi.org/10.1371/journal.pone.0054220*](https://doi.org/10.1371/journal.pone.0054220)*), while T7812G (Gly81Gly) and C9420A (A617A) substitutions are thought to enhance viral replication (*[*https://doi.org/10.3389/fmicb.2022.894200*](https://doi.org/10.3389/fmicb.2022.894200)*). The severe sfRNA (subgenomic flavivirus RNA) mutation (G10296A) may enhance immune evasion by altering interactions with the host factors like TRIM25 (*[*https://doi.org/10.1126/science.aab3369*](https://doi.org/10.1126/science.aab3369)*)”*.

11. The data linking SNVs to disease severity (Page 24, Lines 680–690) are compelling but would benefit from cross-referencing to similar global studies.

We thank the reviewer for the query. While we have not included direct references due to the paucity of similar global studies, we have provided global SNV frequency data for comparison in Figure 6, offering a broader context for understanding SNV distribution in relation to disease severity and also their presence in the global countries. At the same time, we have included those references that are relevant to this work vis-à-vis dengue virus genome SNV and disease severity, in the discussion of the revised manuscript, attaching here for the easy perusal of the reviewer.

*“The genetic variability is known to affect viral virulence and modulate clinical outcomes, emphasizing the need to evaluate its role in the clinical manifestations amongst the dengue patients (*[*https://doi.org/10.1128/Spectrum.00256-21*](https://doi.org/10.1128/Spectrum.00256-21)*). Prior to this study, few investigations have linked SNVs in the E and NS5 regions of DENV-2 to disease severity. For example, an isoleucine-to-valine substitution at position 322 in EDIII of the E gene has been associated with both dengue fever (DF) and dengue hemorrhagic fever (DHF) (*[*https://doi.org/10.1371/journal.pone.0121696*](https://doi.org/10.1371/journal.pone.0121696)*). The mild fNR2 (flaviviral nuclease-resistant RNA) mutation (A10424G) helps maintain sfRNA (subgenomic flavivirus RNA) stability for efficient transmission (*[*https://doi.org/10.1016/j.isci.2019.05.019*](https://doi.org/10.1016/j.isci.2019.05.019)*).”*

12. Add a supplementary table for the SNVs with their genomic positions, allele frequencies, and clinical correlations.

In response to the reviewer’s query, we have provided SNVs along with their genomic positions and clinical correlations in **Supplementary Table 6**. Additionally, we have now included allele frequencies in the same table in the revised manuscript.

13. Consider reordering some subsections for logical flow (e.g., SNV identification before phenotype association).

We thank the reviewer for the suggestion. We have reordered our results section by shifting “**Monophyletic clustering of cosmopolitan DENV-2 genotypes from India”** after **“Significant Alterations in the TLC, Platelets, and Liver Enzymes across DENV-2 Infected-Patients”** and “**Leukopenia, Thrombocytopenia and Elevated Liver Enzymes Differentiates Dengue Severity”**.

14. The conclusion reiterates key findings effectively. However, some statements (Page 28, Lines 720–725) lack nuance, especially concerning the limitations of the study.

We are thankful for the reviewer’s comments. For better understanding of the limitations of the study, the following paragraph is appended here for easy perusal of the reviewer.

***“Limitations of the study***

*While our study provides significant insights into the genomic variability of DENV-2 and its potential association with disease severity sub-phenotypes, few limitations must be acknowledged for future consideration/s. First, the absence of key clinical markers such as cytokine profiling and immune response indicators limits a more comprehensive understanding of host-pathogen interactions. The cross-sectional nature of our study also restricts insights into the temporal evolution of SNVs, highlighting the need for longitudinal analyses across multiple outbreaks. Moreover, as our dataset is derived from a single outbreak in a specific geographic region, future studies incorporating diverse viral strains from multiple endemic regions are essential for broader applicability of these findings. Potential biases in sequencing representation must also be considered, as our dataset primarily includes symptomatic cases, and the inclusion of asymptomatic infections could offer a more complete perspective on viral adaptation and transmission dynamics. Lastly, while our study identifies rare SNVs that may contribute to immune evasion or increased virulence, their precise mechanistic roles in dengue pathogenesis require further research. Addressing these limitations in future studies will provide a more comprehensive understanding of DENV-2 evolution, its clinical implications, and potential mechanisms driving disease severity.”*

15. While potential applications of SNVs in clinical practice are mentioned (Page 29, Lines 730–735), more specific examples would add value.

We acknowledge the reviewer’s suggestion. While we have discussed the potential clinical applications of SNVs, we agree that incorporating more specific examples would enhance the discussion. Based on our findings, certain SNVs associated with clinically severe dengue cases could serve as biomarkers for early risk assessment, aiding in patient stratification and clinical management. However, it is important to note that experimental validation of these SNVs remains limited, and further functional studies and longitudinal clinical data are necessary to establish their clinical relevance. We have highlighted the existing gaps in validation studies in both the Introduction and Discussion sections, emphasizing the need for future research to confirm their significance in dengue pathogenesis.

16. Compare findings with the current state of knowledge to emphasize novel contributions.
    Provide actionable steps for future research or surveillance programs.

We acknowledge the reviewer for the query. Our study provides novel insights into SNV distribution across severity sub-groups, revealing key SNVs in the DENV-2 genome that may influence dengue pathogenesis. Unlike, previous studies that primarily focuses on dengue clinical parameters about prognostic biomarkers of dengue (<https://doi.org/10.1371/journal.pntd.0012846>), demographics of dengue virus where the genotype shifts played a vital role in the epidemiological shifts (<https://doi.org/10.1371/journal.pntd.0004511>) or in the genomic studies which pinpoints the SNVs with the disease severity, lacks the definite number of virus population to compare (<https://doi.org/10.1128/Spectrum.00256-21>). Our findings highlight the integrative analysis of DENV-2 - epidemics, clinical and SNVs in relation to disease severity sub-phenotypes. Future research should investigate the functional impact of these mutations on viral replication, immune evasion, and host interactions, while genomic surveillance programs should integrate these findings to track emerging variants and assess their role in outbreak severity.
