# Supplementary material for: Genomic hotspots in the DENV-2 serotype (E, NS4B, and NS5 genes) are associated with dengue disease severity in the endemic region of India
Source: PLoS Negl Trop Dis. 2025 Apr 29;19(4):e0013034. doi: 10.1371/journal.pntd.0013034 (PMC12040166; doi:10.1371/journal.pntd.0013034)
Supplement: Attachment — Submitted filename: Response_to_Reviewers_auresp_2.docx [file pntd.0013034.s014.docx]

**Response to Reviewer**

Dear Editor and the Reviewers,

Thank for facilitating the journey of our manuscript towards publication and being accessible to the academic community vis-à-vis the Dengue genome plasticity as it infects large number of individuals, especially sub-tropical countries like India.

We have addressed couple of suggestions during this minor revision and hope it would be fit for acceptance thereafter.

**Reviewer #1:**

1. Please revise the added part in the new version of the manuscript (lines 669-673).
   “While SNVs in the 3′UTR and their epidemiological roles are well studied, our findings highlight distinct evolutionary pressures in dengue virus adaptation. The mild fNR2 (flaviviral nuclease resistant RNA) mutation (A10424G) helps maintain sfRNA (subgenomic flavivirus RNA) stability for efficient transmission (<https://doi.org/10.1016/j.isci.2019.05.019>), while the clinically severe sfRNA mutation (G10296A) may enhance immune evasion by altering interactions with the host factors like TRIM25 (<https://doi.org/10.1126/science.aab3369>). This suggests a potential balance between viral fitness and immune adaptation, influencing disease severity.”
   Since there was no functional characterization of the SNVs described to be associated, you may not state "The mild fNR2 mutation helps maintain sfRNA stability… Please rephrase, this could potentially be true, but at the moment, no evidence is shown. Please avoid expressions like ..the mild mutation or "the clinically severe mutation".. It would be scientifically better to write " a variant A or B is associated with mild or severe characteristics of a disease, but mutations themselves cannot be mild or severe.

We thank the reviewer for the valuable suggestion. In alignment with your suggestion and our commitment to scientific accuracy, we have revised the quoted paragraph in the updated revised manuscript. For ease of reference, the revised version is provided below, page 37, line number 681-689,

*“While SNVs in the 3′UTR and their epidemiological roles are well studied, our findings highlight plausible distinct evolutionary pressures in the dengue virus adaptation. Specifically, the statistically significant SNV from 3’UTR, A10424G associated with mild phenotype belong to fNR2 (flavivirus nuclease-resistant RNA) region, could potentially contribute to maintaining sfRNA (subgenomic flavivirus RNA) stability which may play a role in efficient viral transmission (*[*https://doi.org/10.1016/j.isci.2019.05.019*](https://doi.org/10.1016/j.isci.2019.05.019)*) Conversely, other significant SNV G10296A associated with clinically severe phenotype in the sfRNA region, may potentially enhance the immune evasion properties, since the sfRNA region primarily involves in interaction with the host factors like TRIM25 (*[*https://doi.org/10.1126/science.aab3369*](https://doi.org/10.1126/science.aab3369)*). This suggests a potential balance between viral fitness and immune adaptation, influencing disease severity. However, further evidence is required to confirm these potential functional roles of the SNVs identified in our study using clinical patients.”*

Additionally, following your valued suggestion, we have carefully reviewed the whole manuscript and edited the term mild mutation/moderate mutation/clinically severe mutation as a mutation associated with mild, moderate, or clinically severe phenotype throughout the manuscript.

2. Your new finding that the " liver enzyme, AST level observed significantly high in the dengue patients group having thrombocytopenia and both thrombocytopenia and leukopenia, suggesting AST level in dengue patient could potentially be used as the prognostic marker for both thrombocytopenia and leukopenia." should be included in the manuscript.

We are very thankful for your insightful suggestion. In the revised submission, we have included this statement, page 35, line number 636-640,

“*The liver enzyme, AST level, was observed significantly high in the dengue patient’s subgroup having thrombocytopenia and both thrombocytopenia and leukopenia, suggesting AST level in dengue patient could potentially be used as the prognostic marker for both thrombocytopenia and leukopenia."*

**Reviewer #2:**

1. In response to the reviewer's comment, the authors added one paragraph on the effect of mutation on protein function at lines 504-521. However, among the seven mutations in DI-DIII regions, only C2073T changes amino acid. Similarly, among the six mutations in D3 region, only A2105G changes amino acid. Discussion of the effect of synonymous substitutions on protein function is rather ridiculous and need to be corrected.

We acknowledge the reviewer’s perspective. To improve the clarity we have edited the section and updated in the revised submission. The revised paragraph is appended below for your easy perusal, page 27, line number 514-520.

*“The non-synonymous SNVs C2073T and A2105G result in amino acid changes in the viral proteins, while the synonymous SNVs likely affect viral processes through codon usage bias, translation efficiency, transcript stability, and interactions with host factors. Notably, a study by Luciana et al. (*[*https://doi.org/10.1038/s44320-024-00052-7*](https://doi.org/10.1038/s44320-024-00052-7)*) highlights the selection of synonymous mutations that favour DENV's preferred nonoptimal codons, which enhance viral fitness*. *Further targeted studies in this direction would further strengthen these potential functional understanding of the synonymous mutations. Additionally, in the NS4B and NS5 regions, we observe a mixture of SNV associations, suggesting diverse functional impacts on viral replication, immune modulation, and overall disease progression.”*
